# ﻿Taxonomic revision of two dominant *Munidopsis* species (Decapoda, Anomura, Munidopsidae) from the cold seeps in the northern South China Sea: new records and complementary descriptions

**DOI:** 10.3897/zookeys.1261.171276

**Published:** 2025-11-26

**Authors:** Dong Dong, Charlotte A. Seid, Xinzheng Li, Greg W. Rouse

**Affiliations:** 1 Department of Marine Organism Taxonomy & Phylogeny, Qingdao Key Laboratory of Marine Biodiversity and Conservation, Institute of Oceanology, Chinese Academy of Sciences, Qingdao 266071, China Chinese Academy of Sciences Qingdao China; 2 Scripps Institution of Oceanography, University of California San Diego, La Jolla, CA 92093, USA University of California San Diego La Jolla United States of America; 3 University of Chinese Academy of Sciences, Beijing, China University of Chinese Academy of Sciences Beijing China; 4 Laoshan Lab, Qingdao 266237, China Laoshan Lab Qingdao China

**Keywords:** Chemosynthetic habitat, Galatheoidea, hydrothermal vent, squat lobster, taxonomic revision

## Abstract

*Munidopsis* species represent some of the dominant macrobenthos in cold seep habitats of the northern South China Sea. Earlier studies documented high abundances of *M. verrilli* Benedict, 1902 at Site F cold seep and *M. lauensis* Baba & de Saint Laurent, 1992 at Haima cold seep. However, comparative morphological and genetic analysis of additional materials from the deep waters of California, Manus Basin, and southern Okinawa Trough now indicate that those specimens in the South China Sea require taxonomic revision: the specimens at Site F should be *M. longispinosa* Cubelio, Tsuchida & Watanabe, 2007 and the specimens at Haima cold seep belong to *M. ryukyuensis* Cubelio, Tsuchida & Watanabe, 2007. Complementary descriptions of *M. ryukyuensis*, *M. longispinosa* and *M. verrilli*, as well as their geographic distributions, are provided to facilitate future identification.

## ﻿Introduction

*Munidopsis* Whiteaves, 1874 is a genus of squat lobster family Munidopsidae typically inhabiting the deep sea and currently comprising 284 species worldwide (Komai et al. 2025; DecaNet 2025). Members of this genus are common in chemosynthetic environments such as hydrothermal vents and cold seeps. The northwest Pacific hosts numerous back-arc vents and continental marginal seepage, providing exceptional stages for the evolution and speciation of *Munidopsis* species. Two cold seepage fields in the northern South China Sea, Site F and Haima cold seeps, have been investigated extensively in recent years, and several *Munidopsis* species have been reported from these two fields. In Site F, a dominant species *M. verrilli* Benedict, 1902 was recorded within the mussel bed (Dong and Li 2015), whereas *M. lauensis* Baba & de Saint Laurent, 1992 was observed in high abundance at the Haima seep field (Dong et al. 2021). Since their discovery, the two species have drawn significant attention from the academic community, leading to many studies on their genetics, ecology, and biochemistry (e.g. Feng et al. 2023; Wang et al. 2025). Both species are known to have wide distribution ranges within the Indo-Pacific. *Munidopsis lauensis* is endemic to the chemosynthetic habitats from the Brothers Seamount off New Zealand to the middle Indian Ocean and South China Sea (Baba and de Saint Laurent 1992; Cubelio et al. 2007b; Lin et al. 2013; Hwang et al. 2022). In contrast, *M. verrilli* has been recorded inhabiting diverse deep-sea habitats throughout the Pacific Ocean (Baba and Poore 2002; Baba 2005; Komai and Matsuzaki 2016; Rodríguez-Flores 2025). The genetic congruence of *M. lauensis* populations from the Manus Basin, Lau Basin, and Indian Ocean has been well studied (Thaler et al. 2014; Hwang et al. 2022; Rodríguez-Flores et al. 2023); however, *M. verrilli* remains uncharacterized at the molecular level from its type locality, complicating the identification of specimens from other regions, especially given the brevity of the original description on the type specimens. Two additional, closely related species, *M. longispinosa* Cubelio, Tsuchida & Watanabe, 2007 and *M. ryukyuensis* Cubelio, Tsuchida & Watanabe, 2007, were established based on specimens only from the Hatoma Knoll hydrothermal vents in the southern Okinawa Trough. *Munidopsis longispinosa* and *M. ryukyuensis* are morphologically very similar to *M. verrilli* and *M. lauensis*, respectively, with only subtle differences (Cubelio et al. 2007c). Due to the scarcity of reports and the lack of molecular data for *M. longispinosa* and *M. ryukyuensis*, their systematic relationships with respective relatives are still unclear. This further complicates the identification of *Munidopsis* species within the chemosynthetic habitats of the South China Sea.

In the present study, we re-examined material from the two cold seeps in the South China Sea and two hydrothermal vents, Tangyin field and Lion Chimney field, in the southern Okinawa Trough (Suppl. material 1). These two hydrothermal vents fields are geographically adjacent to the Hatoma Knoll (100–150 km) and thus can basically represent the type locality of *M. longispinosa* and *M. ryukyuensis* under the circumstance that the molecular data of specimens from the type locality is unavailable. For comparison, we also examined *M. verrilli* specimens collected from the type locality, off southern California, and specimens of *M. lauensis* from the hydrothermal vents in Manus Basin. Manus Basin is geographically close to the type locality of *M. lauensis* in Lau Basin, and the specimens from the two localities have been proved genetically identical (Thaler et al. 2014). Comparative morphological and genetic analyses revealed that the individuals previously identified as *M. verrilli* in the Site F field should be assigned to *M. longispinosa*, whereas those previously identified as *M. lauensis* in Haima field should belong to *M. ryukyuensis*. However, several key morphological characters of these two species should be revised, and the intra- and interspecific distinctions require re-evaluation. Given the high abundance and ecological importance of these two species in cold seep ecosystems, and in light of the brief original description of *M. verrilli*, here we provide complementary descriptions of all the three species to facilitate future identification. The morphological differences of these species from their relatives are discussed. *Munidopsis longispinosa* and *M. ryukyuensis* were previously known only occurring in the hydrothermal vents of the southern Okinawa Trough. This study represents their first record in the South China Sea and in cold seep environments.

## ﻿Materials and methods

### ﻿Morphological examination

The size of the specimen is given as the postorbital carapace length (**pcl**), which refers to the carapace length excluding the rostrum. The length of rostrum was measured along the midline from the tip to between the bases of mesial eyespines. The specimens from the South China Sea, Tangyin hydrothermal vent and Manus Basin were deposited in the Marine Biology Museum (**MBM**), Chinese Academy of Sciences. The specimens of *M. verrilli* examined were deposited in the Scripps Institution of Oceanography Benthic Invertebrate Collection (**SIO-BIC**). Abbreviations used in the text are: **Mxp3** (third maxilliped), **P1** (first pereopod, cheliped), and **P2–4** (second to fourth pereopods, first to third ambulatory legs).

### ﻿Genetic analysis

Partial sequences of a mitochondrial gene, cytochrome c oxidase subunit I (*COI*), were sequenced for each species to compare the genetic distances and perform the phylogenetic analysis. Total genomic DNA was extracted from pleon muscle tissue using the EasyPure^®^ Marine Animal Genomic DNA Kit (TransGen, Beijing, China). The *COI* gene was amplified via polymerase chain reaction (PCR) by primers HCO2198/LCOgala (Folmer et al. 1994; Dong et al. 2019) following the original process.

PCR products were purified and sequenced in both forward and reverse directions. Contigs were then assembled and verified from bidirectional sequences using the software package SeqMan in DNASTAR Lasergene (DNASTAR, Inc.). To screen for the putative presence of pseudogenes, the obtained sequences were translated to amino acid sequences and checked for the presence of stop codons using ExPASy (http://web.expasy.org/translate). The sequences were aligned using the software Mega 6.06 (Tamura et al. 2013) and then manually trimmed. The genetic distances between species or populations were estimated according to the Kimura 2-parameter (Kimura 1980) model in MEGA 6.06 (Table 2). Phylogenetic relationships were inferred using maximum likelihood (ML) by IQ-Tree (Nguyen et al. 2015). The best nucleotide base substitution model for the alignment was determined by ModelFinder (Kalyaanamoorthy et al. 2017). Nodal supports of ML trees were evaluated by ultrafast bootstrap (UFBoot) with 5000 replicates (Minh et al. 2013). The implementation of ModelFinder and IQ-TREE was pipelined in the program PhyloSuite (Zhang et al. 2020).

Sequences of *M. ryukyuensis*, *M. longispinosa*, and *M. lauensis* from the Manus Basin were generated in the present study. The *M. verrilli* specimens from the SIO-BIC were fixed in formalin solution and unsuitable for molecular study. Fortunately, with the help of Dr. Paula C. Rodríguez-Flores, we obtained the *COI* sequences of specimens which were identified as *M. verrilli* in the National Museum of Natural History, Smithsonian Institution, U.S.A (**USNM**); these specimens were also collected from the type locality, deep sea off the southern California (voucher nos. USNM 1464026 and 1188648). Sequences of *M. asiatica* Marin, 2020 (= *M. similis* Smith, 1885) from the Sea of Okhotsk, *M. similis* from California waters, and *M. lauensis* from the type locality (Hine Hina, Lau Basin) and Indian Ocean were downloaded from the GenBank (Table 1). Two species of another galatheoid family Munididae Ahyong, Baba, Macpherson & Poore, 2010 were chosen as the outgroups (Table 1).

**Table 1.** Molecular data that involved for phylogenetic analysis in this study including vouchers, GenBank accession numbers, sampling localities, and references. *Sadayoshia acropora* and *Grimothea planipes* are designated as outgroups.

Taxa	Vouchers	GenBank No.	Locality	Reference
*M. lauensis*	–	EF157852	Hine Hina, Lau Basin	Cubelio et al. 2007a
*M. lauensis*	HS6	OL958457	Indian Ocean	Hwang et al. 2022
*M. lauensis*	M041	PX111639	Manus Basin	Present study
*M. longispinosa*	MBM287955	PX111642	Tangyin hydrothermal vent, Okinawa Trough	Present study
*M. longispinosa*	MBM287957	PX111641	Site F cold seep, northern South China Sea	Present study
*M. longispinosa*	MBM287956	PX111640	Site F cold seep, northern South China Sea	Present study
*M. ryukyuensis*	MBM287952	PX111638	Lion Chimney hydrothermal vent, Okinawa Trough	Present study
*M. ryukyuensis*	MBM287953_1	PX111635	Haima cold seep, northern South China Sea	Present study
*M. ryukyuensis*	MBM287953_2	PX111636	Haima cold seep, northern South China Sea	Present study
*M. ryukyuensis*	MBM287953_3	PX111637	Haima cold seep, northern South China Sea	Present study
*M. similis*	SIO-BIC-C11171	ON886843	Off southern California	Rodríguez-Flores et al. 2023
*M. similis*	SIO-BIC-C13964	ON886866	Off southern California	Rodríguez-Flores et al. 2023
*M. similis*	MCZ162084	ON886896	Off southern California	Rodríguez-Flores et al. 2023
*M. similis*	MIMB 39449	MN964889	Sea of Okhotsk	Marin 2020
M. cf. verrilli	USNM1188648	–	Off southern California	–
M. cf. verrilli	USNM1464026	–	Off southern California	–
*Sadayoshia acropora*	MNHN-IU-2014-20070	ON886885	–	Rodríguez-Flores et al. 2023
*Grimothea planipes*	MCZ74091-	ON886903	Off southern California	Rodríguez-Flores et al. 2023

**Table 2.** Inter- and intraspecific genetic distances (%) among the five *Munidopsis* species based on the *COI* gene.

	*M. lauensis*	*M. ryukyuensis*	*M. longispinosa*	*M. similis*	M. cf. verrilli
*M. lauensis*	0–0.2				
*M. ryukyuensis*	13.1–13.8	0–0.6			
*M. longispinosa*	17.2	17.2–17.9	0		
*M. similis*	16–17.5	12.7–14.9	12.8–13.2	0.6–2	
M. cf. verrilli	17.5–19.8	14.2–16.3	12.8–14.6	0.8–3.8	1.4

## ﻿Results

### ﻿Phylogenetics

**Figure 1.**
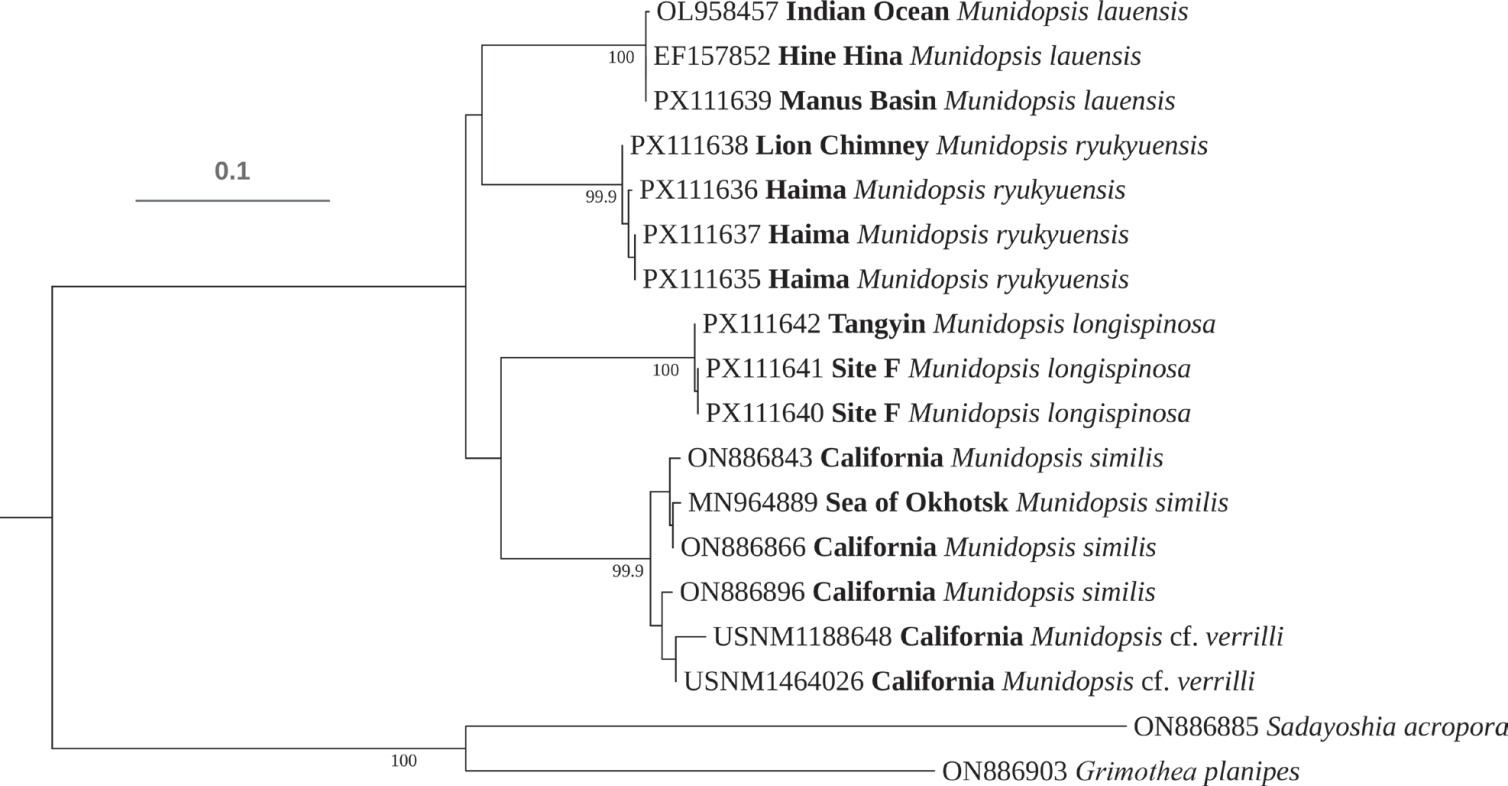
Phylogenetic tree obtained after the maximum likelihood analysis based on the *COI* sequences. UFBoot values (≥ 95%) are indicated adjacent to the nodes. Specimens of *Munidopsis* are labeled with GenBank accession numbers (voucher numbers for M. cf. verrilli) and collection localities (in bold).

Eight new sequences were obtained in this study (GenBank numbers: PX111635–PX111642). According to the phylogenetic tree (Fig. 1), individuals of *M. ryukyuensis* from the Lion Chimney hydrothermal vent and Haima cold seep formed a monophyletic clade, distinct from the *M. lauensis* clade which comprised of specimens from the Lau Basin, Manus Basin, and Indian Ocean. Similarly, individuals of *M. longispinosa* from the Tangyin hydrothermal vent and Site F cold seep clustered together, compared to the clade containing *M. verrilli* and *M. similis* from the East Pacific and the northwest Pacific. All the four clades were highly supported with 100% UFBoot values. It is noteworthy that the UFBoot supports for the internal nodes were insignificant, thereby leaving the phylogenetic relationships among the four clades unresolved in the analysis based solely on the *COI* sequence.

### ﻿Systematic account

#### ﻿Subphylum Crustacea Brünnich, 1772

**Order Decapoda Latreille, 1802**

**Infraorder Anomura MacLeay, 1838**

**Family Munidopsidae Ortmann, 1898**

**Genus *Munidopsis* Whiteaves, 1874**

##### ﻿ Munidopsis ryukyuensis

Taxon classificationAnimaliaDecapodaMunidopsidae

Cubelio, Tsuchida & Watanabe, 2007

912ACF51-9E3E-58E6-9E3E-8A26461BDE60

**Figure 2.**
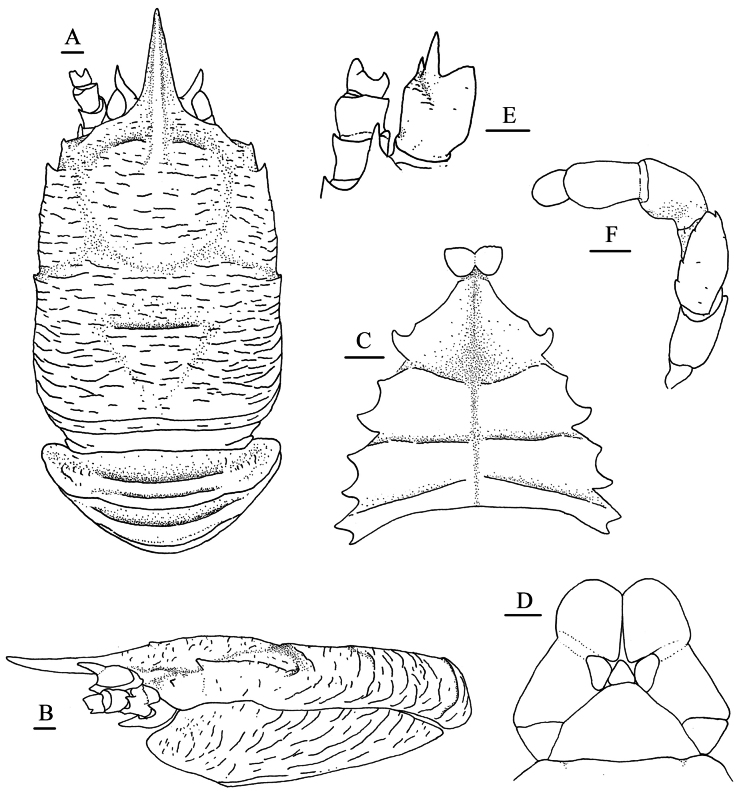
*Munidopsis ryukyuensis* Cubelio, Tsuchida & Watanabe, 2007, MBM287953, Haima cold seep, male, pcl 16.1 mm. **A.** Carapace and pleonal tergites 1–3, dorsal view; **B.** Carapace and left pterygostomian flap, lateral view; **C.** Sternal plastron, ventral view; **D.** Telson; **E.** Right antennular and antennal peduncles, ventral view; **F.** Left Mxp3, ventral view. Scale bars: 1.0 mm.

**Figure 3.**
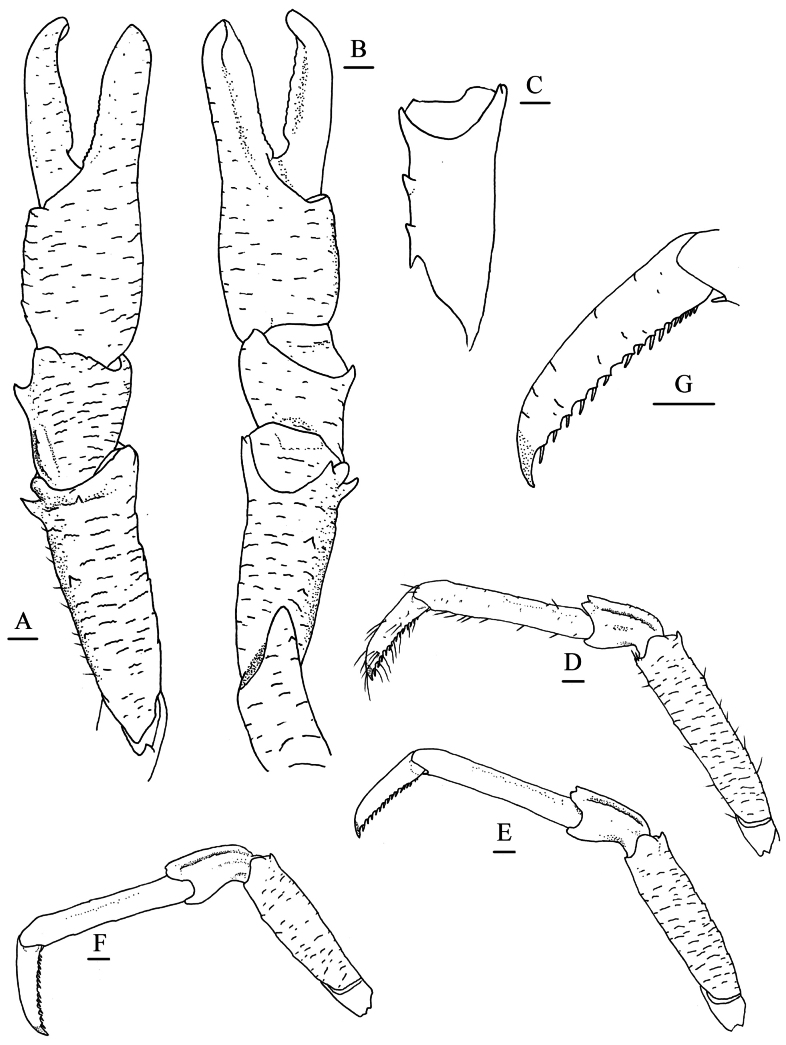
*Munidopsis ryukyuensis* Cubelio, Tsuchida & Watanabe, 2007, MBM287953, Haima cold seep, male, pcl 16.1 mm. **A.** Right P1, setae illustrated only on mesial margin of merus, dorsal view; **B.** Right P1, ventral view; **C.** Left P1 merus, ventral view; **D.** Left P2, setae illustrated, lateral view; **E.** Left P3, lateral view; **F.** Left P4, lateral view; **G.** Dactylus of left P2, lateral view. Scale bars: 1.0 mm.

**Figure 4.**
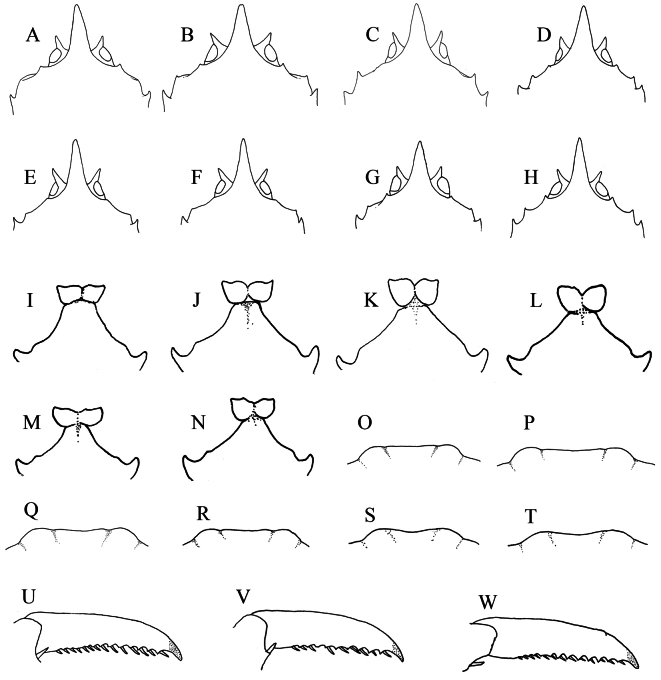
*Munidopsis ryukyuensis* Cubelio, Tsuchida & Watanabe: (**A, B, I, J, O, P, U**) Lion Chimney hydrothermal vent, MBM287952; (**C, D, K, L, Q, R**) Haima cold seep, MBM287953. *Munidopsis lauensis* Baba & de Saint Laurent, 1992: (**E–H**, **M, N, S, T, V, W**) Manus Basin, M041. **A–H.** Anterior part of carapace, epigastric spines omitted, dorsal view; **I–N.** Sternite 3 and 4, ventral view; **O–T.** Posterior margin of pleonal tergite 6; **U–W.** Dactylus of P2, lateral view.

Figs 2, 3, 4A–D, I–L, O–R, U

Munidopsis ryukyuensis Cubelio, Tsuchida & Watanabe, 2007c: 5, figs 3b, 5 (type locality Hatoma Knoll).

###### Material examined.

Southern East China Sea – Okinawa Trough, Lion Chimney hydrothermal vent field • 2 males (17.1–19.7 mm); 24°51'N, 122°42'E; 1374 m depth; 25 Aug. 2016; collected by television grab, R/V Kexue; GenBank no.: PX111638; MBM287952. Northern South China Sea – Haima cold seep field; 16°44'N, 110°28'E • 2 males (16.1–23.8 mm), 1 ovigerous female (14.2 mm); 1387 m depth; 8 Oct. 2024; ROV Faxian of R/V Kexue; GenBank nos: PX111635–PX111637; MBM287953 • 2 females (16.8–21.4 mm), 1 male (16.0 mm); 1380–1390 m depth; 18–19 May 2018; collected by manned submersible Shenhai Yongshi of R/V Tansuo 1; MBM287954.

###### Description.

***Carapace***: distinctly longer than broad (excluding rostrum). Frontal margins oblique, antennal spine absent or reduced as blunt process. Lateral margins subparallel or slightly divergent posteriorly; anterolateral spine small or absent; anterior branchial margin with strong spine at end of anterior cervical groove; posterior branchial margin with short elevated ridge at end of posterior cervical groove. Posterior margin straight, with uninterrupted submarginal ridge. Dorsal surface hairless, covered with numerous short, transverse rugae; rugae longer and more strongly elevated on posterior branchial region; cervical groove deep. Gastric region strongly elevated, with pair of low epigastric ridges. Cardiac region with deep, transverse median groove, posterior cardiac region weakly delineated, subtriangular. Rostrum narrow, triangular and horizontal, ~0.3 remaining carapace length, 2.3× longer than broad; carinate dorsally. Pterygostomial flaps with oblique rugae on surface, anterior end rounded.

***Sternum***: approximately as long as broad, widening posteriorly. Sternite 3 1.9× broader than long, separated into 2 parts by longitudinal median groove; anterior margin weakly serrated, with median notch. Sternite 4 broader than long; anterior margin ~0.5–0.7 width of sternite 3; anterolateral margins oblique; anterior part trapezoidal, with longitudinal median groove. Sternites 5–7 separated by transverse ridges, with shallow groove along midline.

***Pleon***: tergites smooth and unarmed. Tergites 2–4 each with 2 transverse ridges; anterior ridge in tergites 2–4 uninterrupted, posterior ridge in tergite 3 and 4 relatively low and interrupted. Tergite 5 smooth, without distinct groove and ridge. Tergite 6 with straight posterior median margin, with shallow notches on each side. Telson composed of 10 distinct plates.

***Eye***: eyestalk immovable. Peduncle short, broader than cornea. Mesial eyespine short, anterolaterally directed, reaching to proximal ~0.3–0.4 of rostrum. Lateral eyespine absent. Blunt distomesial process present on ventral surface of peduncle. Cornea globular.

***Antennule***: article 1 longer than broad; distal margin with strong ventrolateral spine and small dorsolateral spine; lateral surface slightly inflated, rugose, with oblique shallow groove anteriorly; mesial margin straight.

***Antenna***: peduncle nearly reaching level of proximal 0.3 of rostrum. Article 1 with strong distomesial spine exceeding distal margin of article 2 and small distolateral spine. Article 2 with small distolateral spine. Article 3 subrectangular, unarmed. Article 4 short.

***Mxp3***: ischium shorter than merus, unarmed; crista dentata well-developed. Merus with extensor margin convex, armed with small but distinct disto-extensor spine; flexor margin with 3 small teeth; lateral surface with few rugae. Carpus unarmed. Propodus short, flexor margin gently convex distally.

***P1***: subequal, moderately short, ~1.7× longer than pcl, with short rugae bearing sparse fine setae (those much denser and thicker on larger individuals) on surfaces and margins. Ischium ~0.6 merus length, dorsodistal margin with short spine; ventrodistal margin produced, with 1–3 small subterminal spines sometimes reduced as tubercles. Merus ~0.6 pcl, distally with strong dorsomesial and ventromesial spines and small ventrolateral spine; dorsal surface with small subterminal spine followed by 1 or 2 spines along midline; ventromesial margin unarmed or with 1–3 additional spines on median part. Carpus half of merus length, slightly longer than broad, with strong dorsomesial spine; dorsal surface mesially with longitudinal, rugose ridge (ridge armed with spine on larger individuals). Chela compressed dorsoventrally, palm ~1/2 of merus length, 1.2× longer than broad; lateral and mesial margins rugose; fixed finger rugose on lateral margin. Dactylus 1.2× palm length; occlusal margins weakly crenulate and distally spooned, with blunt process on proximal 1/3.

***P2–4***: surfaces and extensor margins with scale-like rugae and sparse setae on (those much thicker on larger individuals). P2 ~1.8 pcl, reaching base of P1 dactylus. Meri subequal in width and decreasing in length posteriorly, P2 merus 0.7 pcl and 4.4× longer than broad, P3 and P4 meri ~0.9 and 0.8 P2 merus length, respectively; extensor margin with short distal spine and tubercle-like proximal spines; flexor margin rugose, with blunt distal spine. Carpi subequal in length on P2–4, ~0.4 P2 merus length; extensor margin ridged, with small distal spine; lateral surface with low, submarginal ridge along extensor margin ending in short distal spine (P2) or unarmed (P3 and P4). Propodi subequal in length and width on P2–4, 0.9 P2 merus length, and 6.8× longer than broad; extensor margin unarmed; flexor margin with pair of small distal corneous spines only. Dactyli ~1/2 of propodus length; extensor margin proximally straight; distal claw strongly curving; flexor margin straight, with 17–19 movable corneous spines on nearly entire length, each spine of distal 1/2 margin present on low triangular tooth, ultimate spine closer to penultimate spine than to distal claw.

***P1–4*** without epipods.

###### Coloration.

Entirely whitish.

###### Habitat.

Chemosynthetic environments: cold seep and hydrothermal vent.

###### Distribution.

Northeastern South China Sea and Okinawa Trough, southern East China Sea; depth 1374–1487 m.

###### Genetics.

The genetic distance between *M. ryukyuensis* and *M. lauensis* is 13.1%–13.8%. The genetic distances between specimens of *M. ryukyuensis* from the Okinawa Trough and the Haima field are 0.5%–0.6% (Table 2).

###### Remarks.

*Munidopsis ryukyuensis* is morphologically very similar to *M. lauensis* in almost every aspect. Cubelio et al. (2007c) noted that *M. ryukyuensis* differs from *M. lauensis* in having a broadly triangular and straight rostrum, the mesial eyespine more than half the length of the rostrum and directed almost laterally, and a strongly curved distal claw of the P2–4 dactyli. We compared specimens of *M. ryukyuensis* with the material of *M. lauensis* from the Manus Basin and found that the these characters are variable in both species: the length/width ratio of the rostrum in both species ranges from 1.8–2.3, the mesial eyespine only reaches the proximal 0.3–0.4 of rostrum (Fig. 4A–H), and the distal claw of the P2–4 dactyli is strongly curved in most specimens of both species (Fig. 4U–W). Likewise, in the carapace, the antennal spines (situated above the antennal peduncle) and the anterolateral spines vary from acute spines to blunt projection (Fig. 4A–H). In both species, the chelipeds and meri of P2–4 are usually more spinose in males than in females; the males have a row of spines on the ventromesial margin of the P1 merus and a stout distal spine or tubercle on the extensor margin of the P2 merus, whereas these spines are reduced in the females. Larger individuals occasionally bear long setae on the surfaces of P1–4. These variations make the recognition of the two species challenging. Nevertheless, we found some minor but consistent differences based on the specimens examined. In *M. ryukyuensis*, the anterior margin of the sternite 4 is relatively broad, 0.5–0.7 width of the sternite 3 (Fig. 4I–L), and the posteromedian margin of the pleonal tergite 6 is relatively straight with shallow lateral notches (Fig. 4O–R). In contrast, *M. lauensis* usually has a narrower sternite 4 with the anterior margin 0.4–0.5 width of the sternite 3 (Fig. 4M, N), and the posteromedian margin of the pleonal tergite 6 is rather weakly concave (Fig. 4S, T). Baba and de Saint Laurent (1992) also described a narrow sternite 4 in the type material of *M. lauensis*, consistent with our specimens, but illustrated a straight posteromedian margin of pleonal tergite 6, suggesting that this character should be re-examined with more specimens. Notably, *M. lauensis* also occurs at the Site F cold seep field (Lin et al. 2013; Dong and Li 2015). Our observations reveal that the Site F specimens possess a broad sternite 4, resembling that of *M. ryukyuensis*. However, the genetic distance based on the *COI* gene indicates that *M. lauensis* in Site F is genetically consistent with populations from the Manus Basin and Lau Basin, whereas the nuclear gene genes suggest a more complex relationship (unpublished). We therefore temporarily assign the Site F specimens to *M. lauensis*.

##### ﻿ Munidopsis longispinosa

Taxon classificationAnimaliaDecapodaMunidopsidae

Cubelio, Tsuchida & Watanabe, 2007

442BF91E-EB6A-52DF-ADE1-34A5B4ED4F6C

Figs 5, 6

Munidopsis longispinosa Cubelio, Tsuchida & Watanabe, 2007c: 5, figs 3b, 5 (type locality: Hatoma Knoll, Okinawa Trough).
Munidopsis verrilli Dong and Li 2015: 94, figs 3, 6, 8C (not Munidopsis verrilli Benedict, 1902).

###### Material examined.

Southern East China Sea – Okinawa Trough, Tangyin hydrothermal vent field • 1 male (26.4 mm); 25°04'N, 122°34'E; 1219 m depth; 7 May 2014; collected by television grab, R/V Kexue; GenBank no.: PX111642; MBM287955. Northeastern South China Sea – Site F cold seep field; 22°7'N, 119°17'E; 1160 m depth; collected by Faxian ROV of R/V Kexue • 1 female (20.8 mm); 16 Mar. 2014; MBM189174 • 1 female (14.3 mm), 4 males (9.2–23.1 mm), 6 ovigerous females (15.6–29.0 mm); 3 Jun 2020; GenBank no.: PX111640; MBM287956 • 5 males (15.1–32.1 mm); 9 Jun 2021; GenBank no.: PX111641; MBM287957 • 1 male (17.2 mm); Jun 2021; MBM287958.

###### Description.

***Carapace***: distinctly longer than broad (excluding rostrum). Frontal margins oblique, antennal spine well developed. Lateral margins subparallel, sparsely setose; anterolateral spine relatively short, anteriorly directed; anterior branchial margin with 3 (rarely 4) spines; posterior branchial margin with spine at end of posterior cervical groove. Posterior margin slightly concave, with elevated submarginal ridge. Dorsal surface with regions moderately defined, covered with short, transverse rugae; rugae longer and more strongly elevated on posterior branchial region, bearing short setae particularly dense on lateral branchial region; cervical groove deep. Gastric region strongly elevated, with pair of epigastric spines. Cardiac region with deep, transverse median groove, posterior cardiac region slightly elevated. Intestinal region weakly delineated. Rostrum narrowly triangular, nearly horizontal, ~0.2 remaining carapace length, 1.7× longer than broad; dorsal surface smoothly carinate. Pterygostomial flaps with oblique rugae on surface, anterior end blunt.

**Figure 5.**
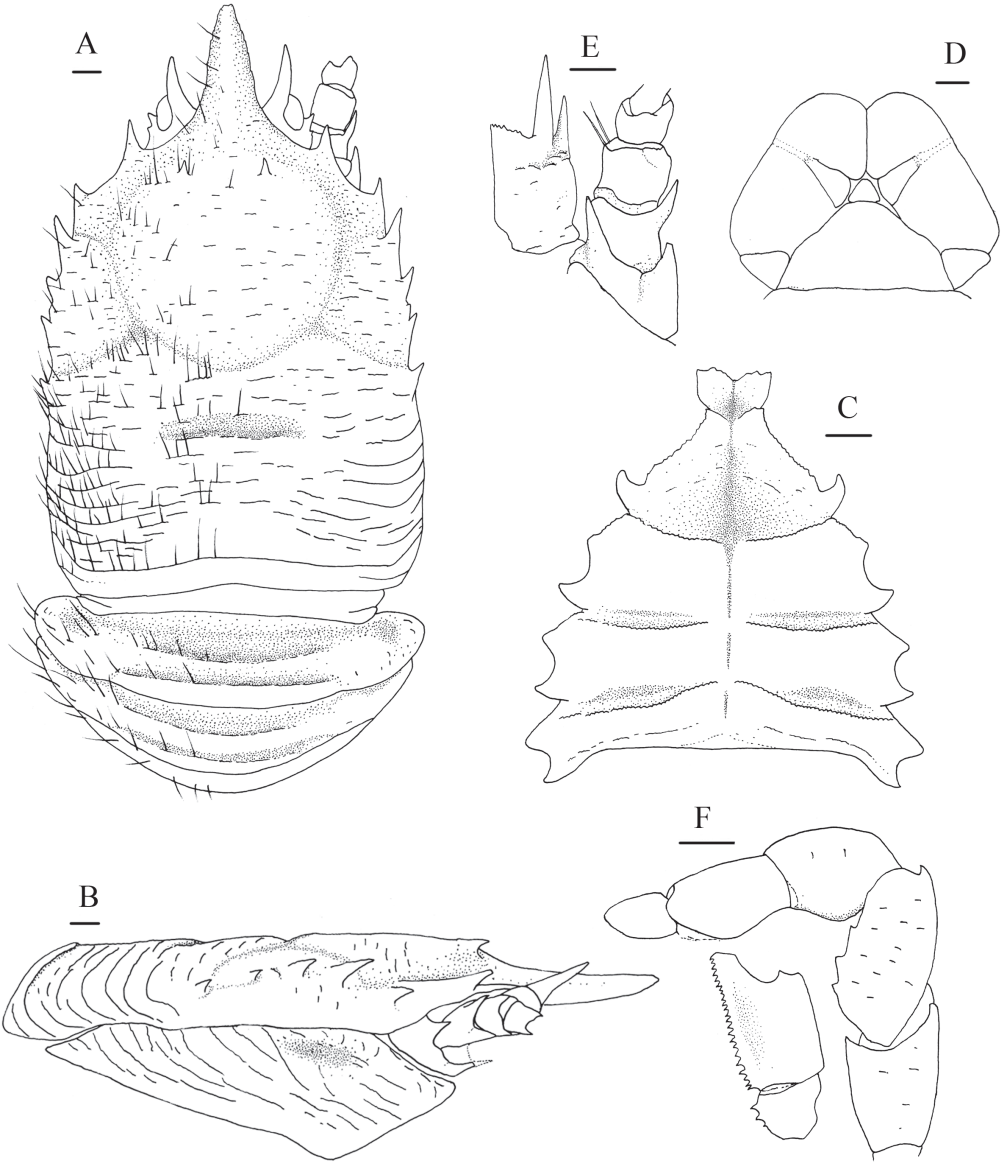
*Munidopsis longispinosa* Cubelio, Tsuchida & Watanabe, 2007, MBM287958, Site F cold seep, male, pcl 17.2 mm. **A.** Carapace and pleonal tergites 1–3, setae illustrated only on left part, dorsal view; **B.** Carapace and right pterygostomian flap, lateral view; **C.** Sternal plastron, ventral view; **D.** Telson; **E.** Left antennular and antennal peduncles, ventral view; **F.** Left Mxp3 with crista dentata, ventral view. Scale bars: 1.0 mm.

***Sternum***: approximately as long as broad, widening posteriorly. Sternite 3 1.7× broader than long, divided into 2 parts by longitudinal median groove; anterior margin with median notch, bearing small lateral tooth. Sternite 4 distinctly broader than long; anterolateral margin denticulate; anterior part narrow, with longitudinal median groove; ventral surface depressed medially and with some short rugae. Sternites 5–7 separated by transverse ridges, with interrupted longitudinal median groove.

**Figure 6.**
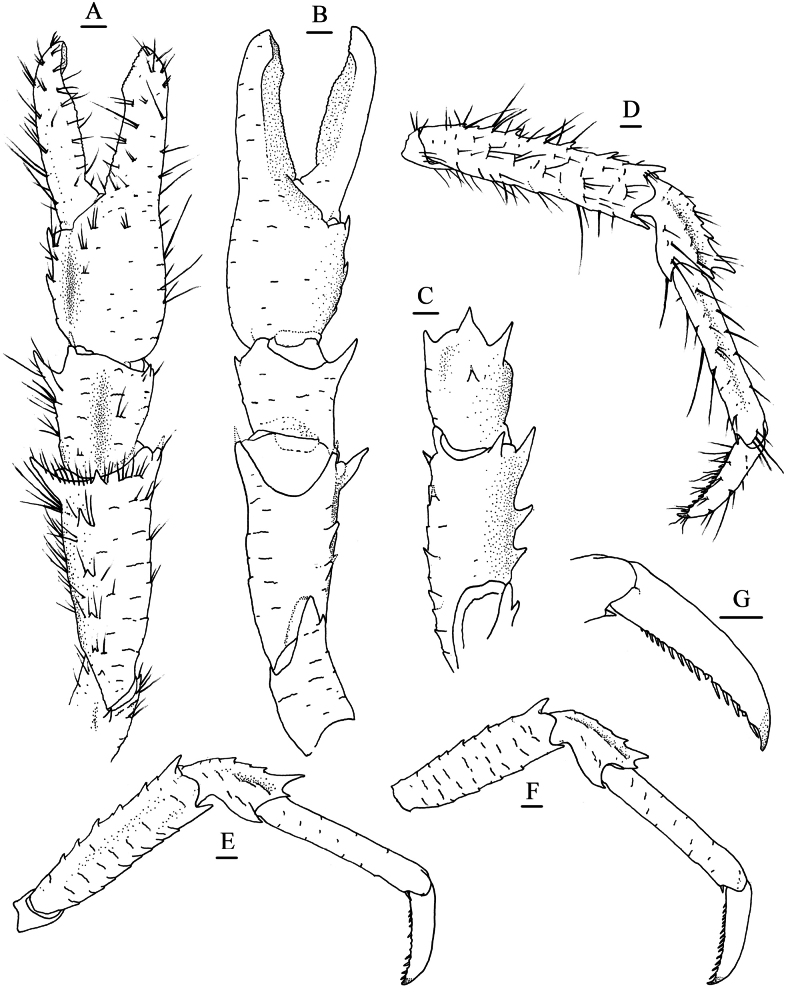
*Munidopsis longispinosa* Cubelio, Tsuchida & Watanabe, 2007, MBM287958, Site F cold seep, male, pcl 17.2 mm. **A.** Right P1, setae illustrated, dorsal view; **B.** Right P1, ventral view; **C.** Left P1 merus and carpus, dorsomesial view; **D.** Right P2, setae illustrated, lateral view; **E.** Right P3, lateral view; **F.** Right P4, lateral view; **G.** Dactylus of right P2, lateral view. Scale bars: 1.0 mm.

***Pleon***: tergites smooth, unarmed. Tergites 2–4 each with 2 transverse ridges bearing dense setae anteriorly, posterior ridge on tergite 4 relatively low and medially interrupted. Tergite 5 smooth, without distinct groove and ridge. Tergite 6 with straight posteromedian margin. Telson composed of 10 distinct plates.

***Eye***: eyestalk immovable. Peduncle short, broader than cornea. Mesial eyespine elongated, anteriorly or anterolaterally directed, reaching to distal 0.4 of rostrum. Lateral eyespine short, sometimes with affiliated smaller spines. Cornea globular.

***Antennule***: article 1 longer than broad; distal margin with strong ventrolateral spine and relatively small dorsolateral spine; lateral surface slightly inflated, with oblique groove extending from base of dorsolateral distal spine to median part of ventral surface; mesial margin straight.

***Antenna***: peduncle thick, nearly reaching to level of midlength of rostrum. Article 1 with strong distomesial spine reaching distal margin of article 2 and short distolateral spines reaching midlength of article 2. Article 2 armed with short distolateral spine reaching midlength of article 3. Article 3 subrectangular, unarmed. Article 4 short.

***Mxp3***: ischium slightly shorter than merus; disto-flexor corner with acute spine; crista dentata well-developed. Merus with extensor margin convex, armed with strong disto-extensor spine; flexor margin with 3 small teeth; lateral surface with sparse short rugae. Carpus with few short rugae on lateral surface. Propodus short, flexor margins convex.

***P1***: subequal, moderately stout, ~1.7× longer than pcl, covered with numerous stiff setae on surfaces and margins. Ischium ~0.6 merus length, dorsodistal margin with acute spine; ventrodistal margin produced, with strong subterminal spine; surfaces covered with short rugae. Merus ~1/2 of pcl, with short, transverse rugae on surfaces; distal margin with strong dorsal, dorsomesial, ventromesial and ventrolateral spines: dorsal spine followed by row of spines along midline of dorsal surface, ventromesial spine followed by 2 strong median spines; mesial surface smooth. Carpus half of merus length, longer than broad; surfaces with short rugae; distal margin with dorsal, dorsomesial, dorsolateral and lateral spines, dorsomesial spine strongest, followed by 1 or 2 spines along dorsomesial margin. Chela compressed dorsoventrally, palm ~0.6 merus length, 1.1× longer than broad, with short rugae on surfaces; lateral margin slightly convex; mesial margin armed with 3 (rarely 1 or 2) spines. Dactylus 1.4× palm length, with tufts of short setae on margins and surfaces; occlusal margins weakly serrated and distally spooned, with low triangular process on distal third of fixed finger.

***P2–4***: slender; margins and surfaces bearing dense long, stiff setae, lateral surface with short scale-like rugae. P2 ~1.9× pcl, reaching base of P1 dactylus. Meri subequal in width on P2–4, decreasing in length posteriorly; P2 merus 0.7× pcl and 4.8× longer than broad, P3 and P4 meri ~0.9 and 0.8 length of P2 merus, respectively; extensor margin armed with row of spines decreasing in size proximally; flexor margin rugose, with strong distal spine. Carpi subequal in length on P2–4, ~0.4 P2 merus length; extensor margin ridged, with strong distal spine; lateral surface with low, submarginal ridge along extensor margin, ending in short distal spine; flexor margin with acute distal spine. Propodi subequal in width on P2–4; P2 propodus subequal or slightly longer than P3 and P4 propodi, 0.8 P2 merus length, and 6.5× longer than broad; extensor margin unarmed; flexor margin distally with pair of small but distinct corneous spines. Dactyli ~1/2 propodus length; extensor proximally straight, distal claw strongly curving; flexor margin straight, with 14 or 15 movable corneous spines on distal 0.8 length, each spine of distal half margin present on low, broad triangular tooth, ultimate spine closer to penultimate spine than to distal claw.

***P1–4*** without epipods.

###### Coloration.

Entirely whitish.

###### Habitat.

Chemosynthetic environments: cold seeps and hydrothermal vents.

###### Distribution.

Northeastern South China Sea and Okinawa Trough, southern East China Sea; depth 1160–1481 m.

###### Genetics.

The genetic distance between *M. longispinosa* and *M. verrilli* from southern California is 12.8%–14.6%. No genetic distances between specimens of *M. longispinosa* from the Okinawa Trough and the Site F site (Table 2).

###### Remarks.

Cubelio et al. (2007c) described *M. longispinosa* based only on the male holotype collected from the hydrothermal-vent field of the Hatoma Knoll in the Okinawa Trough. Our specimens share several key morphological features with the holotype, including the presence of a pair of epigastric spines and five lateral marginal spines of the carapace, and elongated mesial eyespine. However, the original description and figures lacked details on the spination of the P1 merus and carpus. In addition, the holotype was described to have small accessory spines (tubercles) near the epigastric spines, a row of flexor spines on the P2–4 meri, and unarmed flexor margins on the P2–4 propodi (Cubelio et al. 2007c); these characters contrast with our material. Actually, judging from the figures provided by Cubelio et al. (2007c), the holotype seems to lack the accessory gastric spines (tubercles) and the flexor spines on the P2 merus. In our specimens, tuberculate rugae may occur on the gastric region and the flexor margins of the P2–4 meri, but they never form distinct spines. The distal pair of corneous spines on the flexor margin of the P2–4 propodi are exceptionally small and easily overlooked. The holotype and other Hatoma Knoll material are unavailable for further morphological and genetic comparison, but given the general morphological similarity and the close proximity of the Tangyin hydrothermal field to the Hatoma Knoll, we still assign our specimens to *M. longispinosa*. The observed morphological differences from the holotype are likely attributable to intraspecific variation and insufficient original description.

The specimens of *M. longispinosa* from the Site F seep were previously misidentified as *M. verrilli* (Dong and Li 2015) because they both have a pair of epigastric spines only on the carapace dorsal surface, a row of five spines on the carapace lateral margin, unarmed rostrum, mesial and lateral eyespines, and P2 not reaching the distal end of P1. *Munidopsis longispinosa* can be readily distinguished from *M. verrilli* in the following characters: the mesial eyespine is slender and reaches distal ~0.4–0.5 of the rostrum, the lateral cardiac regions of the carapace are ill-defined, the P1 merus has two rows of spines (median spines are absent on the mesial surface), the P1 carpus has four spines on the distal margin (an additional spine is present on the distolateral margin), and the flexor margin of P2–4 propodi has a pair of distal corneous spines only. In *M. verrilli*, the mesial eyespine is short and only reaches the proximal 0.3–0.4 of the rostrum, the lateral cardiac regions of the carapace are elevated, the P1 merus has three rows of spines (distinct median spines are present on the mesial surface), the P1 carpus has three spines on the distal margin (a spine is absent on the distolateral margin), and the flexor margin of P2–4 propodi has three corneous spines including the distal pair. Other minor differences of *M. longispinosa* from *M. verrilli* include thicker, stiffer setae on the P1, three (rarely one or two) instead of usually two spines on the mesial margin of the P1 palm, and small instead of strong spines on the extensor margin of the P2–4 carpi.

Dong and Li (2015) pointed out that the Site F material was different from *M. verrilli* in having six spines on the carapace lateral margin, including the anterolateral spine. However, the number of the spines seems variable: the fourth lateral spine may bear an accessory spine, giving the appearance of six. The shape of the rostrum is likewise variable in *M. longispinosa*, ranging from short and broadly triangular to slender and narrow (somewhat spiniform).

*Munidopsis longispinosa* is also similar to *M. asiatica* from the Sea of Okhotsk, which was proposed as a junior synonym of *M. similis* by Rodríguez-Flores et al. (2023). *Munidopsis longispinosa* is different from the latter in having ill-defined lateral cardiac regions of the carapace, two rows of spines on the P1 merus and relatively smaller spines on the extensor margin of P2–4 carpi (Smith 1885; Marin 2020; Rodríguez-Flores et al. 2023). Additionally, both *M. asiatica* and *M. similis* seem to have three distal spines on the P1 carpus judging from the photos of the specimens (Marin 2020; Rodríguez-Flores et al. 2023). The armature on the flexor margin of the P2–4 propodi is unclear in *M. similis* but it was illustrated distally unarmed in *M. asiatica* (Marin 2020), a character rather unusual in *Munidopsis* species.

*Munidopsis longispinosa* therefore appears endemic to the chemosynthetic habitats in two regions: the Site F cold seep in the northern South China Sea and the hydrothermal fields in the Okinawa Trough, southern East China Sea.

##### ﻿ Munidopsis verrilli

Taxon classificationAnimaliaDecapodaMunidopsidae

Benedict, 1902

FE6E8420-7DCE-57EF-8CF3-5F826BE97E4B

Figs 7, 8

Munidopsis verrilli Benedict 1902: 291, fig. 3 (type locality: off southern California). —Rathbun 1904: 167. —Schmitt 1921: 169, fig. 108. —McCauley 1972: 415 (list). —Luke 1977: 26. —Wicksten 1982: 245 (list). —Wicksten 1989: 316 (list). —Baba and Poore 2002: 245, fig. 10. —Poore 2004: 238, fig. 65k. —Baba 2005: 194, 298. —Osawa and Takeda 2007: 142, fig. 6C, D. —Baba et al. 2009: 275, figs 252, 253. —Komai and Matsuzaki 2016: 103, figs 7, 8.
Munidopsis sp. cf. M. verrilli —Rodríguez-Flores 2025: 21.

###### Material examined.

USA – off California • 9 males (5–17 mm), 6 females (8–13 mm), 4 ovigerous females (13–17 mm); Catalina Basin, MET Sta. 109; 33°11'N, 118°30'W; 1198–1234 m depth; K. Smith and S. Luke leg.; 31 Jan. 1981; collected by 40’ otter trawl of R/V New Horizon; SIO-BIC C4907.

###### Description.

***Carapace***: distinctly longer than broad (excluding rostrum). Frontal margins oblique, antennal spine well developed. Lateral margins divergent posteriorly, broadest at median posterior branchial margin, bearing sparse long setae; anterolateral spine short, anterolaterally directed; anterior branchial margin with 3 spines; posterior branchial margin convex, with spine at end of posterior cervical groove. Posterior margin concave, submarginal ridge elevated. Dorsal surface with regions well defined, covered with transverse, interrupted short rugae; rugae longer and more strongly elevated on posterior branchial region, sparsely bearing short setae on gastric and lateral branchial region; cervical groove deep. Gastric region strongly elevated, with pair of epigastric spines. Cardiac region clearly delineated, lateral and posterior cardiac regions elevated, median transverse groove deep. Intestinal region subtriangular. Rostrum spiniform or narrowly triangular, nearly horizontal, ~0.2 remaining carapace length, 2.5× longer than broad; dorsal surface smoothly carinate, unarmed or rarely with small median spine. Pterygostomial flaps with oblique rugae on surface, anterior end with small spine.

**Figure 7.**
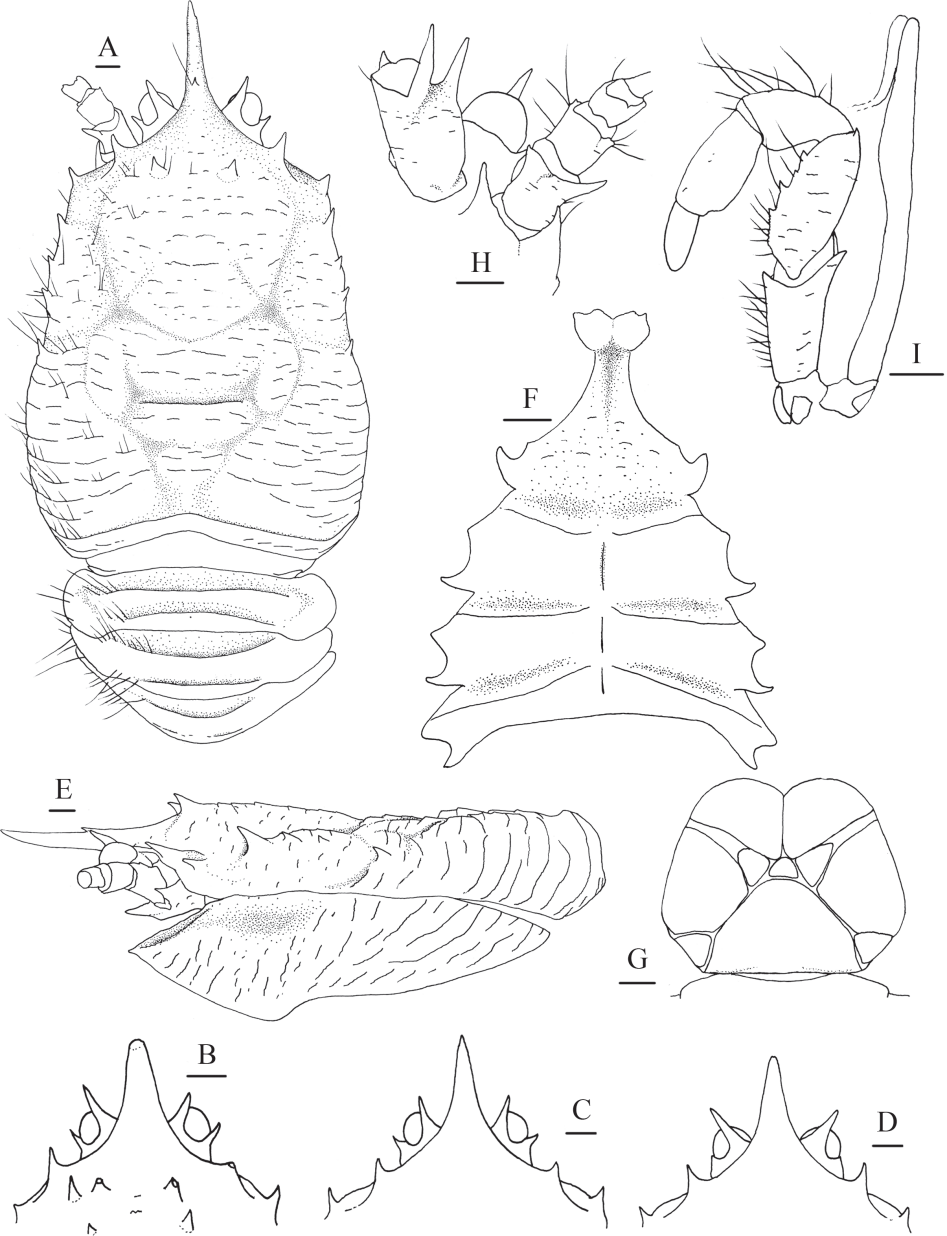
*Munidopsis verrilli* Benedict, 1902, SIO-BIC C4907, Catalina Basin, off California: (**A, E, F, H, I**) male, pcl 17.2 mm; (**B**) male, pcl 10.0 mm; (**C, G**) ovigerous female, pcl 16.5 mm; (**D**) male, pcl 13.0 mm. **A.** Carapace and pleonal tergites 1–3, setae illustrated only on left part, dorsal view; **B–D.** Anterior part of carapace, epigastric spines omitted on (**C**) and (**D**); **E.** Carapace and left pterygostomian flap, lateral view; **F.** Sternal plastron, ventral view; **G.** Telson; **H.** Left antennular and antennal peduncles, ventral view; **I.** Left Mxp3, ventral view. Scale bars: 1.0 mm.

***Sternum***: slightly longer than broad, widening posteriorly. Sternite 3 1.8× broader than long, divided into 2 parts by longitudinal median groove; anterior margin with median notch, bearing small lateral tooth. Sternite 4 slightly broader than long, anterior part with longitudinal median groove, narrowly elongated; posterior part broad, surface depressed and with short scale-like rugae. Sternites 5–7 separated by transverse ridges, with interrupted median groove.

**Figure 8.**
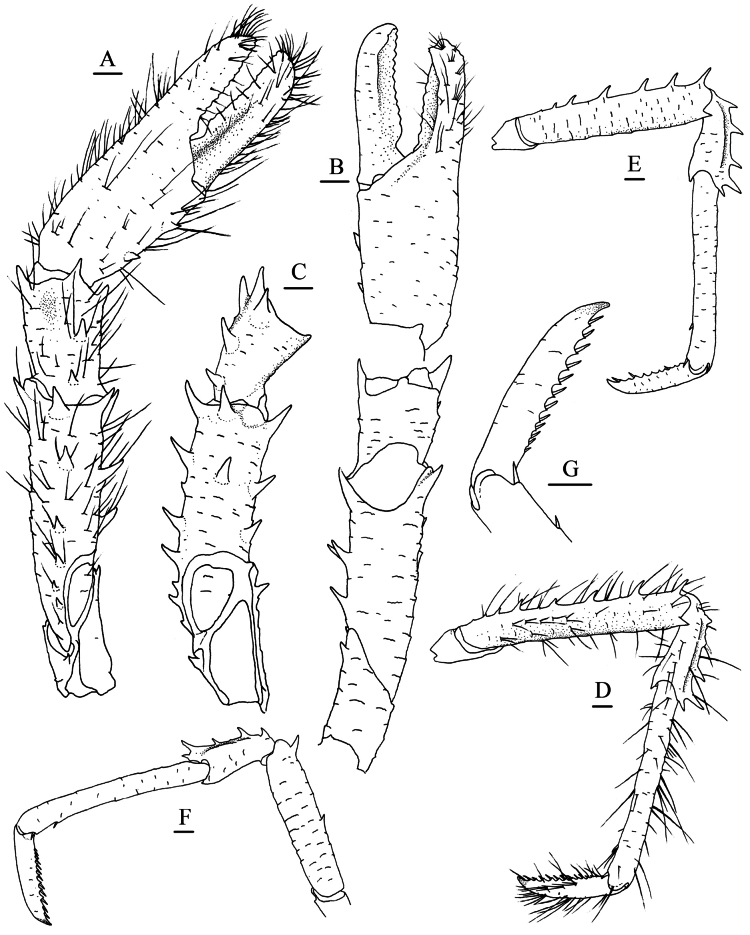
*Munidopsis verrilli* Benedict, 1902, SIO-BIC C4907, Catalina Basin, off California, ovigerous female, pcl 16.5 mm. **A.** Left P1, setae illustrated, dorsal view; **B.** Left P1, ventral view; **C.** Left P1 ischium, merus and carpus, mesial view; **D.** Right P2, setae illustrated, lateral view; **E.** Right P3, lateral view; **F.** Left P4, lateral view; **G.** Dactylus of right P2, lateral view. Scale bars: 1.0 mm.

***Pleon***: tergites smooth and unarmed. Tergite 2–4 each with 2 transverse ridges bearing fine setae anteriorly, posterior ridge on tergite 4 low and interrupted. Tergite 5 smooth, without distinct groove and ridge. Tergite 6 with straight or slightly concave posteromedian margin. Telson composed of 9 or 10 plates.

***Eye***: eyestalk immovable. Peduncle short, broader than cornea. Mesial eyespine relatively long, anteriorly directed, reaching to proximal 0.3–0.4 of rostrum. Lateral eyespine short. Cornea globular.

***Antennule***: article 1 longer than broad; distal margin with strong ventrolateral and dorsolateral spines subequal in size and short mesial spine; lateral surface slightly inflated, with oblique groove extending from base of dorsolateral distal spine to median part of ventral surface; mesial margin straight.

***Antenna***: peduncle thick, reaching to level of midlength of rostrum. Article 1 with distomesial and distolateral spines both reaching midlength of article 2. Article 2 with short distolateral spine reaching midlength of article 3. Article 3 subrectangular, distomesial and distolateral margins minutely denticulate. Article 4 short.

***Mxp3***: ischium slightly shorter than merus, disto-extensor corner with small but acute spine; crista dentata well-developed. Merus with extensor margin slightly convex, rugose, armed with small distal spine; flexor margin denticulate, bearing 3 small spines; lateral surface with short rugae. Carpus with extensor margin slightly rugose. Propodus subrectangular, narrowed distally, extensor and flexor margins subparallel.

***P1***: subequal, 1.5× as long as pcl, densely covered with long setae on surfaces and margins. Ischium short, ~1/2 of merus length; dorsodistal margin armed with acute spine; ventrodistal margin produced, with strong subterminal spine; surfaces covered with short rugae. Merus ~1/2 of pcl; surfaces bearing short transverse rugae; distal margin with strong dorsal, dorsomesial, ventromesial and ventrolateral spines: dorsal spine followed by row of spines along midline of dorsal surface, dorsomesial spine followed by strong median spine on mesial surface, ventromesial spine followed by 2 strong spines on ventral surface. Carpus half of merus length; surfaces with short rugae; distal margin with strong dorsal, dorsolateral and dorsomesial spines; dorsomesial spine followed by row of 2 spines along dorsomesial margin. Chela compressed dorsoventrally, palm 0.6 merus length, 1.6× longer than broad, with short rugae on surfaces; lateral margin straight; mesial margin armed with 2 spines. Fingers approximately as long as palm, thickly setose distally; occlusal margins serrated and distally spooned, with low proximal triangular process on fixed finger.

***P2–4***: slender, with long setae densely on margins and surfaces; lateral surface with short scale-like rugae. P2 ~1.8× pcl, nearly reaching tip of P1 dactylus. Meri slender, decreasing in length posteriorly, P2 merus 0.6 pcl, P3 and P4 merus ~0.8 and 0.7 P2 merus length, respectively; P2 merus ~7.1× longer than broad (length/width ratio on P3 and P4 meri, 5 and 4.5, respectively); extensor margin armed with row of spines decreasing in size proximally; flexor margin rugose, with strong distal spine. Carpi somewhat decreasing in length posteriorly, subequal in width, P2 carpus ~0.4 length of P2 merus, P3 and P4 carpi 0.9 and 0.8 length of P2 carpus, respectively; extensor margin with 2 longitudinal ridges, mesial ridge usually with 4 spines, lateral ridge with relatively small distal spine; flexor margin with minute distal spine. Propodi slender, subequal in width on P2–4; P2 propodus 0.9 length of P2 merus, 9.0× longer than broad, P3 and P4 propodi 0.9 length of P2 propodus; extensor margin slightly rugose, unarmed; flexor margin with pair of distal corneous spines preceded by corneous spine located on distal 0.2. Dactyli 0.4 or 0.5 length of propodus; extensor margin slightly rugose, proximally straight, distal claw curving; flexor margin straight, with 10–12 movable corneous spines on distal 0.8 length, each spine present on low triangular tooth, ultimate spine closer to penultimate spine than to distal claw.

***P1–4*** without epipods.

###### Habitat.

Clay (Baba 2005) and manganese-crust bottom (Rodríguez-Flores 2025).

###### Distribution.

West Pacific: south to Australia and Indonesia and north to Japan; Central Pacific: Johnston Atoll; Eastern Pacific: from off Oregon to southern California; depth 732–4169 m.

###### Remarks.

Baba (2005) noted that the holotype of *M. verrilli* has a denticulate carina on the distolateral margin of the P1 fixed finger, while such feature is absent in our specimens. Baba (2005) regarded this character as an intraspecific variation within this species, because it was also missing in other specimens he examined from the type locality.

Baba and Poore (2002) reported a female from Tasmania, Australia. The diagnosis and figures of their specimens showed three rows of spines on the P1 merus including a median spine on the mesial surface, and three spines along the flexor margin of the P2 and P3 propodi. These characters match the specimens examined of *M. verrilli* from California waters. A single male from the Makassar Strait exhibits the similar traits (Baba 2005). Meanwhile, the key interspecific characters, such as the elevated lateral cardiac region of the carapace, short mesial eyespine, median spine on the mesial surface of P1 merus and a row of strong spines on the extensor margin of P2–3 carpi, can be observed from the photos of specimens from the Okinawa Trough, northeast of Taiwan Island and Nemuro Strait (Osawa and Takeda 2007; Baba et al. 2009; Komai and Matsuzaki 2016), indicating that these West-Pacific specimens also belong to *M. verrilli*.

*Munidopsis verrilli* appears independent of chemosynthetic environments. The California specimens have been taken from mud bottom by otter trawl or beam trawl (Luke 1977). In the Makassar Strait, it was collected from clay bottom (Baba 2005) and in Johnston Atoll, it was obtained on manganese-crust bottom (Rodríguez-Flores 2025). Osawa and Takeda (2007) obtained the specimen by beam trawl in the Okinawa Trough, while Komai and Matsuzaki (2016) captured the specimens with commercial gill net. Given the wide distribution range, molecular methods are needed to confirm the taxonomic consistency and genetic connectivity among populations in different localities.

Rodríguez-Flores et al. (2023) reported *M. similis* from deep waters off southern California, which is identical to the type locality of *M. verrilli*. Their *COI* data show only 0.8–3.8% genetic distances from M. cf. verrilli (USNM 1464026 and 1188648), suggesting a very low divergence (Rodríguez-Flores et al. 2023). *Munidopsis similis* ranges from the West Atlantic to the West Pacific and exhibits low genetic divergence among populations (Rodríguez-Flores et al. 2023). In this case, the taxonomic validity of *M. verrilli* may be questionable. Further molecular evidence of *M. similis*, particularly from the topotypic material collected off New England, is essential to resolve the systematic relationship between the two species.

## Supplementary Material

XML Treatment for Munidopsis ryukyuensis

XML Treatment for Munidopsis longispinosa

XML Treatment for Munidopsis verrilli

## ﻿Additional information

### Use of AI

No use of AI was reported.

### Author ORCIDs

Dong Dong https://orcid.org/0000-0002-8087-7763

Charlotte A. Seid https://orcid.org/0000-0002-5307-691X

Xinzheng Li https://orcid.org/0000-0001-6344-6542

Greg W. Rouse https://orcid.org/0000-0001-9036-9263

### Data availability

All of the data that support the findings of this study are available in the main text or Supplementary Information.

## Supplementary materials

Supplementary material 1 Locations Data type tif Explanation note Locations of the four chemosynthetic sites (red) from which the examined specimens of *Munidopsis ryukyuensis* Cubelio, Tsuchida & Watanabe, 2007 and *Munidopsis longispinosa* Cubelio, Tsuchida & Watanabe, 2007 were collected, with the type locality Hatoma Knoll (blue). https://binary.pensoft.net/file/1478348This dataset is made available under the Open Database License (http://opendatacommons.org/licenses/odbl/1.0/). The Open Database License (ODbL) is a license agreement intended to allow users to freely share, modify, and use this Dataset while maintaining this same freedom for others, provided that the original source and author(s) are credited.Dong Dong, Charlotte A. Seid, Xinzheng Li, Greg W. Rouse
