# Supplementary figures and images for: ﻿Taxonomic revision of two dominant Munidopsis species (Decapoda, Anomura, Munidopsidae) from the cold seeps in the northern South China Sea: new records and complementary descriptions
Source: Zookeys. 2025 Nov 26;1261:165–88. doi: 10.3897/zookeys.1261.171276 (PMC12676479; doi:10.3897/zookeys.1261.171276)

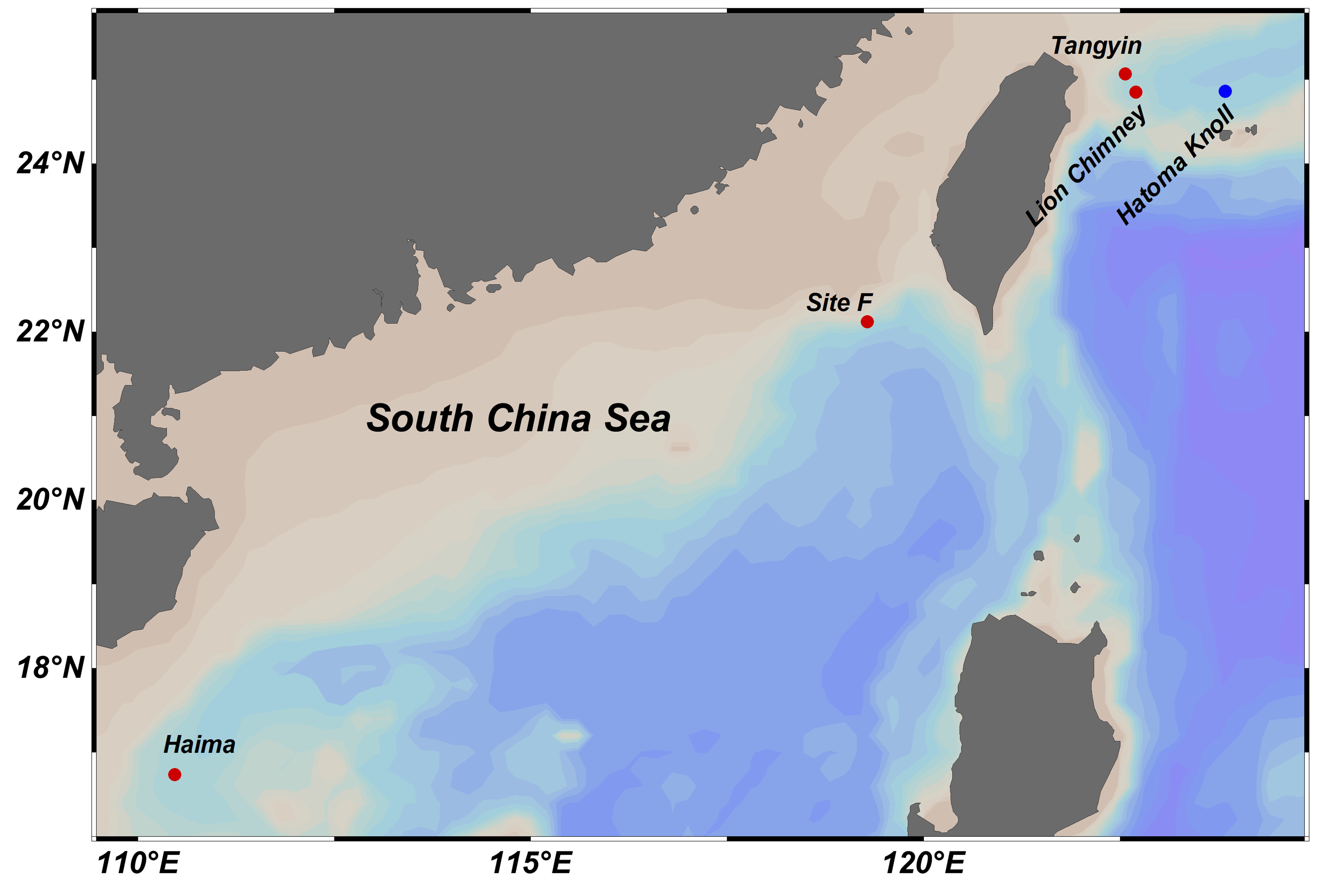

Supplement: Supplementary material 1 — Locations [file zookeys-1261-165_article-171276__-s001.tif]
